# Serum metabolic profiling of patients with diabetic kidney disease based on gas chromatography-mass spectrometry

**DOI:** 10.3389/fmolb.2025.1541440

**Published:** 2025-03-17

**Authors:** Xueyan Bian, Chenwen Wang, Majie Wang, Ailing Yin, Jiayan Xu, Mijia Liu, Hui Wang, Yating Cao, Xin Huang, Chenxue Qin, Ye Zhang, Heming Yu

**Affiliations:** ^1^ Department of Nephrology, The First Affiliated Hospital of Ningbo University, Ningbo, Zhejiang, China; ^2^ State Key Laboratory of Natural Medicines, School of Traditional Chinese Pharmacy, China Pharmaceutical University, Nanjing, China; ^3^ Department of Psychiatry, Affiliated Kangning Hospital of Ningbo University, Ningbo, Zhejiang, China; ^4^ Department of Psychiatry, Ningbo Kangning Hospital, Ningbo, Zhejiang, China; ^5^ Nanjing Hospital of Chinese Medicine Affiliated to Nanjing University of Chinese Medicine, Nanjing, China

**Keywords:** diabetic kidney disease, metabolomics, gas chromatography mass spectrometry, biomarker, myoinositol, gluconic acid

## Abstract

**Introduction:**

Given the increasing incidence rate of diabetic kidney disease (DKD), there is an urgent need for methods to diagnose and treat DKD in clinics.

**Methods:**

Serum samples were collected from 56 DKD patients and 32 healthy controls (HCs) at the First Affiliated Hospital of Ningbo University, and the metabolic profiles were obtained through untargeted metabolomics using gas chromatography mass spectrometry. The data were then analyzed using principal components analysis, orthogonal partial least-squares discriminant analysis, Pearson correlation analysis, and receiver operating characteristic (ROC) curve.

**Results:**

It was found that the serum metabolic profiles of the DKD patients were significantly different from those of the HCs. A total of 68 potential differential metabolites were identified that were involved in arginine biosynthesis, ascorbate and aldarate metabolism, and galactose metabolism, among others; a total of 31 differential metabolites were also identified between early-stage (EDG) and late-stage (LDG) DKD patients. Additionally, 30 significant metabolic differences were observed among the EDG, LDG, and HC groups. Based on Pearson correlation analysis between the abundances of the differential metabolites and clinical markers (estimated glomerular filtration rate, blood urea nitrogen, serum creatinine, and urinary albumin/creatinine ratio) and area under the ROC curve (AUROC) analysis, the AUROC values of myoinositol and gluconic acid were found to be 0.992 and 0.991, respectively, which can be used to distinguish DKD patients from HCs.

**Discussion:**

These results indicate that myoinositol and gluconic acid could possibly be used as biomarkers of DKD.

## 1 Introduction

Diabetic kidney disease (DKD) is common among 40% of patients with diabetes and results in the fastest growth of chronic kidney disease (CKD) along with its associated morbidity and mortality (Rayego-Mateos et al., 2023; Wu et al., 2022). Globally, the number of DKD patients is expected to increase proportionally with an increase in global diabetes prevalence, eventually affecting approximately 780 million people by 2045 (Limonte et al., 2022). The pathogenesis of DKD is multifactorial, and its initiation and progression involve many metabolic, hemodynamic, inflammatory, and other pathophysiological processes (Falkevall et al., 2017; Ricciardi and Gnudi, 2021). Clinically, glomerular hyperfiltration is regarded as the initial stage of DKD; at this stage, patients typically exhibit no obvious pathological symptoms. As the glomerular basement membrane thickens and mesangial hyperplasia progresses, patients begin to develop albuminuria, signaling the onset of the microalbuminuria stage. As the disease develops, the presence of tubulointerstitial fibrosis can lead to further increase in albuminuria, eventually progressing to macroalbuminuria. In the later stages, when more than 50% of the glomeruli undergo sclerosis, DKD progresses to its advanced phase and is often accompanied by complications, such as retinopathy, atherosclerosis, and microangiopathy (Tuttle et al., 2014; Qi et al., 2017). At present, the clinical diagnosis of DKD is based on the level of urinary albumin, which is generally characterized by persistent albuminuria with a urinary albumin/creatinine ratio (UACR) exceeding 30 mg/g and accompanied by decreased renal function (Selby and Taal, 2020); the estimated glomerular filtration rate (eGFR) and renal biopsy are also usually considered during such diagnosis (Oshima et al., 2021). Some studies have reported additional clinical biomarkers that may be useful for diagnosing DKD, including glomerular biomarkers (e.g., cystatin C), tubular biomarkers (e.g., urinary cystatin C, and kidney injury molecule-1), inflammatory biomarkers (e.g., tumor necrotic factor-alpha and transforming growth factor-beta (TGFβ)), oxidative stress biomarkers (e.g., 8-oxo-7,8-dihydro-2′-deoxyguanosine and uric acid), emerging biomarkers (e.g., microRNAs), and certain genetic markers (e.g., aldo-keto reductase family 1 member A1 gene). However, these biomarkers have only moderately enhanced the diagnostic capabilities of the currently available methods. Despite these advancements, the eGFR and UACR remain the cornerstone biomarkers for diagnosing and distinguishing DKD subtypes in clinical practice (Swaminathan et al., 2023; Li et al., 2024). The characteristics of UACR and eGFR are not unique to DKD, and studies have shown that inflammation and fat mass can also affect the eGFR. Kidney biopsies can only be used in certain cases and cannot be used to detect the early stages of DKD (Doshi and Friedman, 2017; Jung and Yoo, 2022; Clingan et al., 2023). Research has demonstrated that targeted interventions and effective prevention during the early stages of DKD can delay progression to renal failure while enhancing patient outcomes. Therefore, more sensitive and specific biomarkers are needed to screen and distinguish DKD patients.

Metabolomics can be applied to the core biological fluids of clinical nephrology diagnosis and prognosis methods, namely, blood and urine (Dubin and Rhee, 2020). It involves measurement of low-weight intermediates and small end products of biochemical processes in biological fluids using mass spectrometry and proton nuclear magnetic resonance spectroscopy, which can be used to identify the biomarkers of DKD and type 2 diabetes (T2D) (Hocher and Adamski, 2017; Jung and Yoo, 2022; Pereira et al., 2022). A urine multiomics platform combining metabolomics and proteomics has been reported previously and used to investigate the biological changes in the pathogenesis of DKD (Jiang et al., 2023). In addition, some metabolites in the urine and serum, such as glucose, lactate, carnosine, leucine, and phenyl acetate, have also been shown to be associated with cancer-related cachexia (Yang et al., 2018).

**FIGURE 1:**
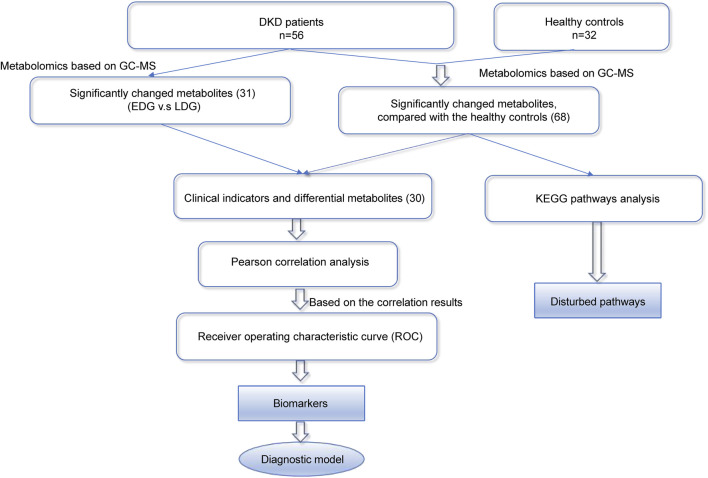
Overall strategy for the discovery of diagnostic and therapeutic biomarkers of diabetic kidney disease (DKD).

Given the frequent occurrence of DKD in the general population (Chen et al., 2023), we intend to use collected clinical serum samples for metabolomics research on disease development using gas chromatography mass spectrometry (GC-MS); accordingly, we performed KEGG pathway analysis, Pearson correlation analysis, and receiver operating characteristic (ROC) curve analysis on the data. Additionally, we conducted differential analysis of the early (EDG) and late (LDG) stages of DKD using metabolomics to provide valuable insights into potential diagnostic biomarkers while enhancing our understanding of the underlying pathophysiology. Thus, two biomarkers were screened based on our findings and are expected to be beneficial for the clinical diagnosis and treatment of DKD (Figure 1).

## 2 Materials and methods

### 2.1 Sample collection

Serum samples were collected from 56 DKD patients and 32 healthy controls (HCs) at the First Affiliated Hospital of Ningbo University. The research was conducted in accordance with the guidelines of both of the Declarations of Helsinki. All study procedures and protocols were approved by the Clinical Trial Ethics Committee for Drugs and Medical Devices of the First Affiliated Hospital of Ningbo University (2021-R144). DKD patients were selected from patients with T2D; the selection criteria included age, gender, body mass index (BMI), eGFR, UACR, and other indicators as well as clinical staging diagnosis results (including hyperfiltration, microalbuminuria, massive albuminuria, and renal failure), while excluding interference from other diseases. The patients were selected on the basis of similarities in age and gender, and the diagnoses were based on clinical indicators encompassing all stages of DKD as comprehensively as possible. The HC group consisted of individuals with normal blood glucose levels and no history of diabetes or other diseases; they were as closely matched as possible to the members of the DKD group in terms of age, gender, and BMI. All samples were collected from the DKD patients and HCs after 8 h of fasting to minimize any potential interference from dietary intake. Informed consent was obtained from each patient or subject after providing a full explanation of the purpose and nature of all procedures used in the study. The samples were collected and stored in a freezer at −80°C until the start of the experiments (Bi et al., 2024).

### 2.2 Sample preparation

Aliquots of 50 μL of the serum were precipitated with 200 μL of methanol (containing 12.5 μg/mL of 1,2-13C myristic acid), followed by vortex mixing for 3 min before being centrifuged at 18,894.2*g* for 10 min at 4°C. The supernatant (100 μL) was then evaporated in a centrifuge concentrator (45°C, 15 kPa). The residue was derived by the addition of 30 μL of methoxypyridine solution (10 mg/mL, 1.5 h at 30°C), followed by trimethylsilyl derivatization using 30 μL of BSTFA (containing 1% TMS, 0.5 h at 37°C). After cooling to room temperature, the mixture was centrifuged at 13,000 rpm for 10 min; then, approximately 40 μL of the supernatant was transferred to a sample vial for analysis. Moreover, 5 µL of each sample was pooled to obtain a quality control (QC) sample for testing during the analysis (Yan et al., 2023).

### 2.3 GC-MS conditions

The samples were analyzed using a GC-MS system (Trace1310/TSQ8000, Thermo, United States). Separation was achieved on a TG-5MS capillary column (250 mm × 30 mm × 0.25 μm, Thermo). The initial oven temperature for gas chromatography (GC) was set to 60°C for 1 min, followed by increasing at the rate of 20°C/min to 320°C and holding for 5 min. The injection volume was 1 μL. The temperature of the inlet and ion source were set to 250°C and 280°C, respectively. Electron impact ionization (70 eV) at the examined m/z range of 50–500 was used (Yang et al., 2022).

### 2.4 Data analysis

Identification of the metabolomics data was performed using MS-DIAL 4.9 software and the NIST library (Zhang et al., 2024a). The raw spectrum files generated by Xcalibur software were processed for peak extraction, deconvolution, compound identification, and peak alignment before being exported as .txt files containing the metabolite information (including metabolite names, retention times, and m/z). All sample data were uploaded to MetaboAnalyst 6.0, initially normalized using the median value, and transformed logarithmically (lg) for further normalization. The final normalized data were introduced to the SIMCA-P 14.0 software package for multivariate statistical analysis. The variables were discriminated using principal component analysis (PCA) and orthogonal partial least-squares discriminant analysis (OPLS-DA) (Thysell et al., 2012). The significance of each metabolite was analyzed through the Mann–Whitney–Wilcoxon test along with false discovery rate (FDR) correction via the Benjamini–Hochberg method. The differential metabolites were screened on the basis of variable importance in projection (VIP) > 1.0 obtained from the OPLS-DA and adjusted p-values (*p*) of < 0.05. MetaboAnalyst 6.0 was used for the KEGG pathway enrichment analysis, and Pearson correlation analysis was conducted between the clinical information and differential metabolites using Origin 2024. Lastly, we used GraphPad Prism 9.5.0 for the ROC analysis (Xiong et al., 2024; Zhang et al., 2024b).

## 3 Results

### 3.1 Clinical sample information

The baseline characteristics and clinical staging information of the DKD patients and HCs included in the metabolomics analysis are presented in Tables 1, 2. These patients were only taking medications specific to DKD, such as metformin hydrochloride. DKD is known to be more prevalent in men, and a higher BMI value not only increases the risk of developing the condition but also contributes to lower eGFR (Clotet-Freixas et al., 2024; Russo et al., 2018). Additionally, the prevalence of DKD increases with age (Iqbal et al., 2024). There were significant differences in various serum indicators between the DKD patients and HCs, including eGFR, albumin, serum creatinine (SCr), and UACR values. These differences were markedly higher for eGFR and UACR, where the former reflects the normality of renal function and the latter reflects the amount of urinary albumin; both of these metrics can indicate the severity of kidney disease.

**TABLE 1:** Clinical sample information.

	Average ± SD		Standard reference interval
	DKD	HC	
Gender (F/M)	18/38	15/17	—
Age (years)	63.33 ± 11.43	50.33 ± 2.52	—
Body mass index (BMI; kg/m^2^)	24.11 ± 3.60	22.74 ± 2.1	—
Hemoglobin A1c (mmol/mol)	119.70 ± 22.97	—	20–42
Estimated glomerular filtration rate (eGFR; mL/min/1.73 m^2^)	45.36 ± 35.90	—	>90
Albumin (g/L)	35.51 ± 5.48	—	40–55
Blood urea nitrogen (BUN; mg/dL)	11.85 ± 6.89	—	7–20
Serum creatinine (μmol/L)	325.29 ± 333.14	—	44–133
Glucose (mmol/L)	7.69 ± 4.41	—	4.4–6.1
Urine creatinine (μmol/L)	8,080.74 ± 9,618.11	—	8,840–17,680
Urinary albumin/creatinine ratio (UACR; mg/g)	1,816.88 ± 2,561.62	—	<30

**TABLE 2:** Clinical sample information for different stages of DKD.

	Average ± SD	
	EDG	LDG
Gender (F/M)	11/21	7/17
Age (years)	63.69 ± 9.86	61.86 ± 14.64
BMI (kg/m^2^)	24.52 ± 3.68	23.69 ± 3.39
Hemoglobin A1c (mmol/mol)	128.47 ± 23.49	104.67 ± 15.40
eGFR (mL/min/1.73 m^2^)	67.05 ± 27.65	8.72 ± 4.86
Albumin (g/L)	36.01 ± 5.95	34.63 ± 4.91
BUN (mg/dL)	9.03 ± 5.46	16.62 ± 6.51
Serum creatinine (μmol/L)	111.25 ± 62.15	692.17 ± 298.97
Glucose (mmol/L)	7.03 ± 4.70	8.84 ± 3.81
Urine creatinine (μmol/L)	6,751.72 ± 3,675.94	10,718.92 ± 15,341.12
UACR (mg/g)	1,139.64 ± 1,952.17	2,990.90 ± 3,164.90

### 3.2 Serum metabolic differences between DKD patients and HCs

**FIGURE 2:**
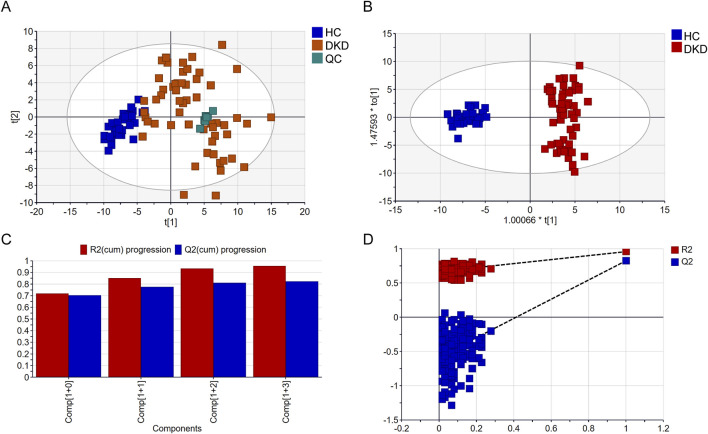
Comparison of serum metabolomics findings between DKD and healthy controls (HCs) based on gas chromatography mass spectrometry (GC-MS): **(A)** scatter plot of DKD patients and HCs in the principal component analysis (PCA) model; **(B)** scatter plot of DKD patients and HCs in the orthogonal partial least-squares discriminant analysis (OPLS-DA) model; **(C)** cross-validation plot; **(D)** permutation test repeated 200 times.

We performed metabolomics analysis of the serum samples from the 56 DKD patients and 32 HCs using a GC-MS system (Supplementary Figure S1), and a total of 147 metabolites were detected. Multivariate data analysis was further performed to obtain the metabolic differences between DKD patients and HCs. The score plot of PCA is shown in Figure 2A; here, the serum samples of the DKD patients were obviously separated from those of the HCs, indicating that there were obvious differences in serum metabolism between the two groups. The clustering of the QC group samples further indicated that our instrument was relatively stable. The supervised classification model OPLS-DA was subsequently used for further analysis. As shown in Figure 2B, the DKD and HC groups can be classified into two categories. The high R^2^Y (cum) = 0.957 and Q^2^ (cum) = 0.824 values demonstrate the strong explanatory and predictive capabilities of this model, respectively (Figure 2C). To prevent overfitting of the original model, permutation tests were performed with 200 iterations (Figure 2D). The R^2^ and Q^2^ values of the original OPLS-DA model were substantially higher than the corresponding values of the permuted models, indicating that the model is not overfitted. The intercepts of R^2^ and Q^2^ at 0.667 and −0.578, respectively, further confirm that the model was reliable and not overfitted (Huang et al., 2023).

**FIGURE 3:**
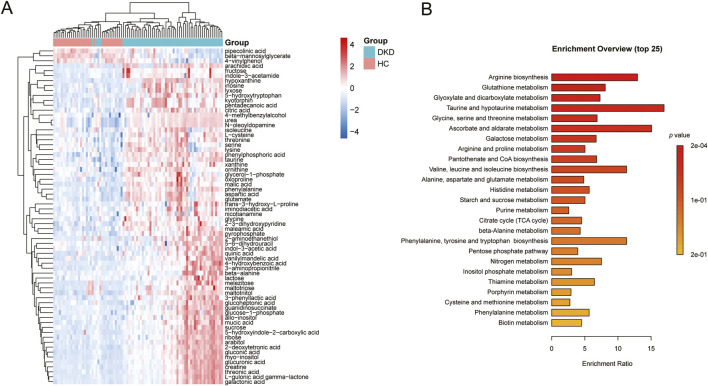
**(A)** Heatmap of the differential metabolites between DKD patients and HCs. **(B)** Metabolic pathways associated with the differential metabolites.

VIP represents the variable weight value of the OPLS-DA model and indicates the contribution of a variable to the variance explanation. Based on the OPLS-DA results, the metabolites were selected based on VIP scores >1 and FDR adjusted *p*-values (FDR-*p*) < 0.05. Thus, a total of 68 differential metabolites were finally obtained (Table 3; Figure 3A). Among these differential metabolites, the carbohydrate compounds and their derivatives include sucrose, arabitol, melezitose, ribose, mucic acid, lactose, glucose-1-phosphate, glucuronic acid, gluconic acid, maltotriose, and glucoheptonic acid; additionally, lyxose and fructose are significantly elevated in DKD. Next, the amino acids and their derivatives, including glutamate, aspartic acid, L-cysteine, oxoproline, 5-hydroxytryptophan, glycine, taurine, threonine, ornithine, trans-3-hydroxy-L-proline, lysine, isoleucine, beta-alanine, serine, creatine, and kyotorphin, also showed higher serum levels in DKD patients than HCs. The other differential metabolites include indole compounds like indole-3-acetamide and indole-3-acetic acid; purine compounds such as inosine, hypoxanthine, and xanthine; and vitamin B derivatives like nicotianamine and myoinositol. Long-chain fatty acids, including pentadecanoic acid and arachidic acid, as well as uremic toxins such as guanidinosuccinate, organic acids like 5-hydroxyindole-2-carboxylic acid, pyrophosphate, and the pyrimidine compound 5-6-dihydrouracil, were found to be elevated in DKD patients compared to HCs. In contrast, the phenolic compound 4-vinylphenol was observed to be higher in the HCs.

**TABLE 3:** Differential metabolites for comparison between the HCs and DKD patients.

Metabolites	Rt/min	*m/z*	VIP	FDR-*p*	*p*	Fold change	Peak area		Chemical formula	CVQC
							HC	DKD		
N-Oleoyldopamine	6.44	148.04	1.59	6.15E-16	7.78E-18	1.08	7.36 ± 0.30	7.96 ± 0.20	C_26_H_43_NO_3_	0.72%
Gluconic acid	11.44	333.17	1.61	6.15E-16	8.37E-18	1.18	5.31 ± 0.13	6.26 ± 0.48	C_6_H_12_O_7_	0.49%
Urea	6.42	146.92	1.56	1.52E-15	3.10E-17	1.06	8.17 ± 0.28	8.69 ± 0.17	CH_4_N_2_O	0.60%
Myoinositol	11.90	305.13	1.60	1.55E-15	4.21E-17	1.12	6.82 ± 0.11	7.64 ± 0.42	C_6_H_12_O_6_	0.22%
2-Deoxytetronic acid	7.83	233.14	1.61	1.89E-15	6.44E-17	1.11	4.84 ± 0.10	5.38 ± 0.27	C_4_H_8_O_4_	0.43%
Maltotriitol	15.05	361.13	1.42	2.62E-13	1.07E-14	1.18	4.87 ± 0.44	5.76 ± 0.40	C_18_H_34_O_16_	1.89%
Phenylphosphoric acid	10.48	287.24	1.47	4.63E-13	2.20E-14	1.02	7.07 ± 0.06	7.21 ± 0.07	C_6_H_7_O_4_P	0.92%
Glutamate	9.10	246.14	1.52	9.88E-13	5.38E-14	1.09	6.73 ± 0.20	7.32 ± 0.30	C_5_H_9_NO_4_	1.54%
Aspartic acid	8.45	232.12	1.50	3.34E-12	2.04E-13	1.08	6.40 ± 0.18	6.92 ± 0.28	C_4_H_7_NO_4_	1.05%
Malic acid	8.23	233.13	1.43	5.40E-12	3.67E-13	1.10	5.15 ± 0.11	5.69 ± 0.29	C_4_H_6_O_5_	0.51%
2-3-Dihydroxypyridine	7.62	240.13	1.31	9.60E-12	7.33E-13	1.02	5.50 ± 0.06	5.63 ± 0.08	C_5_H_5_NO_2_	0.64%
Phenylalanine	6.54	299.64	1.49	9.60E-12	7.83E-13	1.07	6.57 ± 0.17	7.01 ± 0.26	C_9_H_11_NO_2_	1.97%
Maleamic acid	9.17	244.07	1.32	2.73E-11	2.57E-12	1.08	5.35 ± 0.20	5.76 ± 0.24	C_4_H_5_NO_3_	1.98%
L-Gluonic acid gamma-lactone	9.34	217.10	1.49	2.73E-11	2.94E-12	1.18	4.98 ± 0.08	5.88 ± 0.64	C_6_H_10_O_6_	1.21%
Galactonic acid	10.14	292.16	1.49	2.73E-11	2.96E-12	1.17	4.94 ± 0.13	5.78 ± 0.58	C_6_H_12_O_7_	1.34%
3-Phenyllactic acid	8.96	193.12	1.33	2.73E-11	2.97E-12	1.17	4.33 ± 0.19	5.05 ± 0.49	C_9_H_10_O_3_	2.30%
Kyotorphin	9.72	242.20	1.36	4.14E-11	4.79E-12	1.13	4.25 ± 0.19	4.80 ± 0.38	C_15_H_23_N_5_O_4_	6.33%
5-Hydroxyindole-2-carboxylic acid	10.27	231.07	1.45	4.89E-11	5.99E-12	1.19	4.07 ± 0.33	4.86 ± 0.52	C_9_H_7_NO_3_	12.53%
3-Aminopropionitrile	7.72	239.13	1.43	5.70E-11	7.36E-12	1.07	4.43 ± 0.07	4.76 ± 0.23	C_3_H_6_N_2_	1.81%
Creatine	8.75	329.19	1.48	6.66E-11	9.06E-12	1.12	5.87 ± 0.14	6.60 ± 0.53	C_4_H_9_N_3_O_2_	0.97%
Threonic acid	8.62	292.15	1.49	1.09E-10	1.57E-11	1.13	5.79 ± 0.15	6.53 ± 0.52	C_4_H_8_O_5_	0.53%
β-Mannosylglycerate	10.55	217.11	1.31	1.09E-10	1.63E-11	1.14	4.28 ± 0.16	4.87 ± 0.73	C_9_H_16_O_9_	10.75%
Arabitol	9.77	217.12	1.45	2.20E-10	3.44E-11	1.12	5.88 ± 0.15	6.58 ± 0.52	C_5_H_12_O_5_	1.26%
Glucuronic acid	11.08	333.17	1.46	2.35E-10	3.84E-11	1.17	5.57 ± 0.12	6.49 ± 0.69	C_6_H_10_O_7_	0.52%
Ribose	9.68	307.20	1.43	3.50E-10	5.95E-11	1.11	4.46 ± 0.13	4.93 ± 0.34	C_5_H_10_O_5_	0.80%
Mucic acid	11.18	318.18	1.37	5.86E-10	1.04E-10	1.21	4.63 ± 0.30	5.58 ± 0.75	C_6_H_10_O_8_	0.36%
Xanthine	11.56	353.15	1.28	6.67E-10	1.24E-10	1.21	4.07 ± 0.39	4.91 ± 0.57	C_5_H_4_N_4_O_2_	11.12%
Hypoxanthine	10.43	265.17	1.29	6.67E-10	1.27E-10	1.39	3.88 ± 0.19	5.41 ± 1.04	C_5_H_4_N_4_O	18.85%
Melezitose	15.55	361.21	1.26	9.78E-10	1.93E-10	1.23	4.00 ± 0.41	4.93 ± 0.70	C_18_H_32_O_16_	1.09%
4-Hydroxybenzoic acid	9.27	223.12	1.39	1.10E-09	2.25E-10	1.17	4.28 ± 0.11	5.00 ± 0.58	C_7_H_6_O_3_	8.11%
Pentadecanoic acid	9.72	242.20	1.29	1.13E-09	2.39E-10	1.14	4.18 ± 0.19	4.76 ± 0.42	C_15_H_30_O_2_	8.11%
Sucrose	14.41	361.19	1.40	1.20E-09	2.62E-10	1.14	5.41 ± 0.21	6.16 ± 0.59	C_12_H_22_O_11_	0.48%
Indole-3-acetamide	11.33	415.21	1.25	1.62E-09	3.65E-10	1.03	7.09 ± 0.31	7.30 ± 0.32	C_10_H_10_N_2_O	15.66%
Inosine	14.06	217.11	1.20	1.89E-09	4.37E-10	1.21	4.64 ± 0.30	5.63 ± 0.80	C_10_H_12_N_4_O_5_	2.73%
Pyrophosphate	9.44	451.13	1.30	6.35E-09	1.51E-09	1.08	5.91 ± 0.23	6.41 ± 0.37	C_8_H_20_O_7_P_2_	5.71%
Glucose-1-phosphate	13.03	217.10	1.32	7.19E-09	1.76E-09	1.16	5.29 ± 0.32	6.13 ± 0.67	C_6_H_13_O_9_P	1.86%
L-Cysteine	8.71	220.14	1.29	1.61E-08	4.06E-09	1.04	6.22 ± 0.15	6.47 ± 0.18	C_3_H_7_NO_2_S	0.49%
Oxoproline	8.56	156.07	1.32	1.65E-08	4.26E-09	1.06	6.20 ± 0.15	6.55 ± 0.25	C_5_H_7_NO_3_	4.62%
Pipecolinic acid	7.11	156.14	1.24	3.51E-08	9.31E-09	−1.15	5.11 ± 0.28	4.45 ± 0.53	C_6_H_11_NO_2_	7.64%
Arachidic acid	11.48	116.70	1.17	4.86E-08	1.32E-08	1.29	4.32 ± 0.59	5.59 ± 1.11	C_20_H_40_O_2_	6.85%
5-Hydroxytryptophan	9.45	290.21	1.15	5.37E-08	1.50E-08	1.17	3.88 ± 0.21	4.53 ± 0.55	C_11_H_12_N_2_O_3_	4.54%
Alloinositol	11.56	318.20	1.32	6.54E-08	1.87E-08	1.12	5.11 ± 0.18	5.70 ± 0.48	C_6_H_12_O_6_	0.15%
Maltotriose	14.15	204.08	1.22	7.34E-08	2.15E-08	1.12	4.30 ± 0.24	4.82 ± 0.46	C_18_H_32_O_16_	7.47%
Vanillylmandelic acid	10.71	297.16	1.26	7.88E-08	2.36E-08	1.13	4.43 ± 0.33	5.00 ± 0.48	C_9_H_10_O_5_	2.65%
Glucoheptonic acid	10.11	217.14	1.29	1.29E-07	3.95E-08	1.09	5.18 ± 0.18	5.64 ± 0.41	C_7_H_14_O_8_	1.32%
Lyxose	9.50	217.10	1.13	3.13E-07	9.79E-08	1.10	5.76 ± 0.26	6.31 ± 0.51	C_5_H_10_O_5_	1.09%
Nicotianamine	8.94	186.08	1.15	4.38E-07	1.40E-07	1.10	5.54 ± 0.29	6.09 ± 0.47	C_12_H_21_N_3_O_6_	0.88%
Fructose	8.64	263.20	1.10	4.45E-07	1.45E-07	1.06	6.03 ± 0.07	6.37 ± 0.35	C_6_H_12_O_6_	2.79%
4-Methylbenzylalcohol	6.43	189.03	1.17	4.68E-07	1.56E-07	1.06	8.14 ± 0.38	8.61 ± 0.26	C_8_H_10_O	0.11%
Glycine	6.91	174.09	1.12	1.04E-06	3.52E-07	1.05	7.24 ± 0.24	7.63 ± 0.32	C_2_H_5_NO_2_	1.47%
2-Aminoethanethiol	6.31	146.83	1.06	2.15E-06	7.47E-07	1.22	6.00 ± 0.75	7.30 ± 1.32	C_2_H_7_NS	17.08%
Citric acid	10.34	273.12	1.09	2.58E-06	9.12E-07	1.03	6.91 ± 0.12	7.15 ± 0.20	C_6_H_8_O_7_	0.70%
4-Vinylphenol	7.23	192.15	1.11	2.75E-06	9.91E-07	−1.02	6.07 ± 0.06	5.97 ± 0.09	C_8_H_8_O	2.47%
Taurine	9.53	326.15	1.17	4.04E-06	1.49E-06	1.15	5.00 ± 0.45	5.73 ± 0.56	C_2_H_7_NO_3_S	13.84%
Iminodiacetic acid	8.14	232.13	1.12	4.71E-06	1.76E-06	1.06	6.07 ± 0.17	6.46 ± 0.38	C_4_H_7_NO_4_	2.66%
Guanidinosuccinate	11.46	444.19	1.27	4.80E-06	1.83E-06	1.22	3.58 ± 0.18	4.38 ± 0.81	C_5_H_9_N_3_O_4_	4.78%
Threonine	7.50	218.13	1.21	5.59E-06	2.17E-06	1.03	7.17 ± 0.16	7.37 ± 0.18	C_4_H_9_NO_3_	0.49%
Ornithine	9.98	174.15	1.16	1.36E-05	5.35E-06	1.07	6.18 ± 0.36	6.59 ± 0.34	C_5_H_12_N_2_O_2_	1.05%
Glycerol-1-phosphate	10.01	299.10	1.12	3.51E-05	1.43E-05	1.04	5.72 ± 0.17	5.97 ± 0.25	C_3_H_7_O_6_P^-2^	1.23%
Quinic acid	10.61	345.21	1.11	4.55E-05	1.98E-05	1.14	4.55 ± 0.51	5.17 ± 0.74	C_7_H_12_O_6_	0.49%
Trans-3-hydroxy-L-proline	10.09	274.13	1.11	4.70E-05	2.08E-05	1.09	5.18 ± 0.18	5.67 ± 0.56	C_5_H_9_NO_3_	2.31%
Indole-3-acetic acid	11.33	202.16	1.06	6.64E-05	2.98E-05	1.07	4.94 ± 0.27	5.27 ± 0.36	C_10_H_9_NO_2_	1.87%
Lysine	10.90	317.25	1.14	1.34E-04	6.19E-05	1.03	6.71 ± 0.15	6.94 ± 0.28	C_6_H_14_N_2_O_2_	4.38%
Isoleucine	6.77	218.10	1.06	2.21E-04	1.07E-04	1.03	6.67 ± 0.16	6.84 ± 0.19	C_6_H_13_NO_2_	0.57%
β-Alanine	6.46	146.05	1.02	6.65E-04	3.26E-04	1.06	5.52 ± 0.55	5.84 ± 0.31	C_3_H_7_NO_2_	5.00%
Lactose	14.70	204.11	1.04	9.49E-04	4.78E-04	1.07	5.01 ± 0.17	5.36 ± 0.49	C_12_H_22_O_11_	2.75%
Serine	7.30	204.12	1.13	1.31E-03	6.93E-04	1.02	7.37 ± 0.18	7.53 ± 0.20	C_3_H_7_NO_3_	0.49%
5-6-Dihydrouracil	3.96	171.04	1.02	5.59E-03	3.27E-03	1.03	8.09 ± 0.18	8.37 ± 0.48	C_4_H_6_N_2_O_2_	9.09%

Fold change: DKD/HC when DKD > HC; -HC/DKD when HC > DKD.

KEGG analysis of data in the MetPA database (part of MetaboAnalyst) revealed the unique metabolic pathways between the DKD and HC groups (Figure 3B). The most influential metabolic pathways had *p* < 0.05. These include arginine biosynthesis; glutathione metabolism; glyoxylate and dicarboxylate metabolism; taurine and hypotaurine metabolism; glycine, serine, and threonine metabolism; ascorbate and aldarate metabolism; galactose metabolism; arginine and proline metabolism; pantothenate and CoA biosynthesis; valine, leucine, and isoleucine biosynthesis; alanine, aspartate, and glutamate metabolism; and histidine metabolism. Among these, the metabolic pathways for arginine biosynthesis, taurine and hypotaurine metabolism, ascorbate and aldarate metabolism, as well as valine, leucine, and isoleucine biosynthesis were overexpressed. These pathways may thus be useful for studying the prevention, development, and treatment of DKD.

### 3.3 Serum metabolite profiling of the different stages of DKD

**FIGURE 4:**
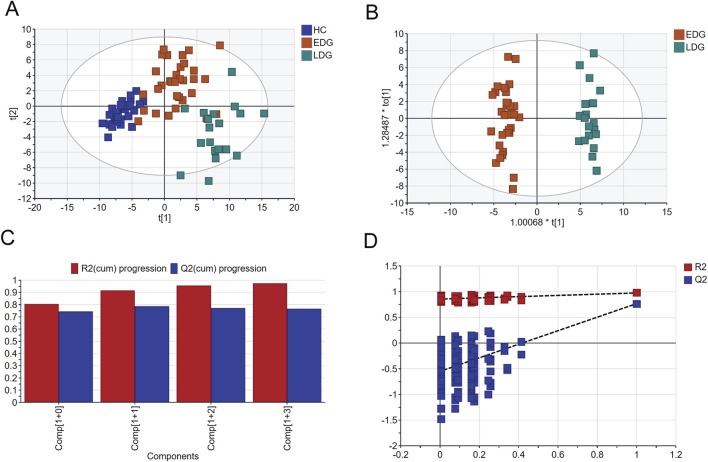
Comparison of serum metabolomics between early-stage (EDG) and late-stage (LDG) patients based on GC-MS: **(A)** scatter plot of EDG and LDG patients based on PCA; **(B)** scatter plot of EDG and LDG patients based on OPLS-DA; **(C)** cross-validation plot; **(D)** permutation test repeated 200 times.

**FIGURE 5:**
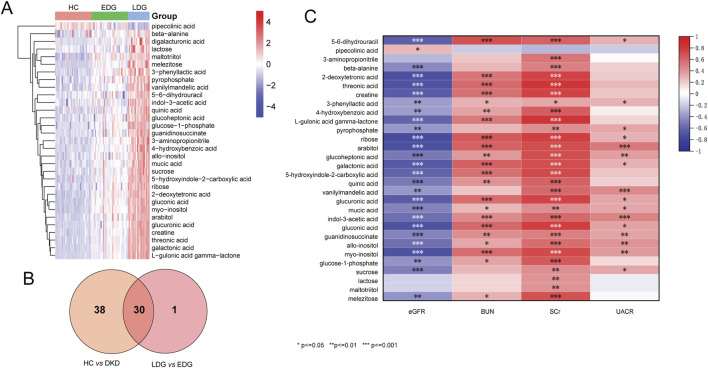
**(A)** Heatmap of the differential metabolites between EDG and LDG patients. **(B)** Venn diagram to determine the common differential metabolites between DKD vs. HC groups and EDG vs. LDG patients. **(C)** Map of the correlation coefficients between the metabolites and four clinical parameters (*p < 0.05, **p < 0.01, ***p < 0.001).

The score scatterplots of the PCA models show clear differentiation among the EDG, LDG, and HC groups (Figure 4A). Furthermore, OPLS-DA was used to differentiate the EDG and LDG patients (Figure 4B). The high R^2^Y (cum) = 0.975 and Q^2^ (cum) = 0.767 values demonstrate the strong explanatory and predictive capabilities of this model (Figure 4C). The results of cross-validation and 200 iterations of the permutation experiment (R^2^ = 0.865, Q^2^ = −0.55) indicate that the models were not overfitted (Figure 4D). Based on the OPLS-DA results, a total of 31 differential metabolites were screened (Table 4; Figure 5A). Among these, there were 30 overlapping differential metabolites between the HC and DKD groups (Figure 5B). Regarding these differential metabolites, carbohydrate compounds and derivatives, such as sucrose, arabitol, melezitose, ribose, mucic acid, lactose, glucose-1-phosphate, glucuronic acid, glucoheptonic acid, and gluconic acid, were elevated in the LDG patients. Additionally, the amino acids and their derivatives, including beta-alanine and creatine, showed significantly higher levels in the LDG patients. Other metabolites with higher levels in the LDG patients included indole-3-acetic acid (a derivative of indole), the vitamin B compound myoinositol, and the uremic toxin guanidinosuccinate. Elevated levels of the organic acid 5-hydroxyindole-2-carboxylic acid, pyrophosphate, the pyrimidine 5-6-dihydrouracil, and the aromatic compound vanillylmandelic acid were also observed in the LDG patients compared to the other two groups. We used Pearson correlation analysis to demonstrate the correlations between the 30 differential metabolites and clinical indicators from the blood tests of the patients (Figure 5C); the results indicated that 13 metabolites were significantly correlated with the four indicators (eGFR, blood urea nitrogen (BUN), SCr, and UACR): alloinositol, myoinositol, gluconic acid, glucuronic acid, galactonic acid, 3-phenyllactic acid, arabitol, mucic acid, ribose, glucoheptonic acid, guanidinosuccinate, indole-3-acetic acid, and 5,6-dihydrouracil.

**TABLE 4:** Differential metabolites for comparisons between the LDG and EDG patients.

Metabolites	Rt/min	*m/z*	VIP	FDR-*p*	*p*	Fold change	Peak area		Chemical formula	CVQC
							EDG	LDG		
L-Gluonic acid gamma-lactone	9.34	217.1	1.95	3.99E-15	2.72E-17	1.21	5.45 ± 0.30	6.61 ± 0.33	C_6_H_10_O_6_	1.21%
Threonic acid	8.62	292.15	1.89	6.23E-13	8.67E-15	1.14	6.20 ± 0.31	7.09 ± 0.22	C_4_H_8_O_5_	0.53%
Creatine	8.75	329.19	1.89	6.23E-13	1.27E-14	1.14	6.27 ± 0.31	7.17 ± 0.25	C_4_H_9_N_3_O_2_	0.97%
5-Hydroxyindole-2-carboxylic acid	10.27	231.07	1.85	1.02E-12	2.79E-14	1.20	4.53 ± 0.32	5.42 ± 0.21	C_9_H_7_NO_3_	12.53%
Galactonic acid	10.14	292.16	1.88	1.99E-12	6.76E-14	1.18	5.41 ± 0.34	6.39 ± 0.31	C_6_H_12_O_7_	1.34%
Myoinositol	11.9	305.13	1.82	8.30E-11	3.68E-12	1.09	7.38 ± 0.26	8.08 ± 0.26	C_6_H_12_O_6_	0.22%
Gluconic acid	11.44	333.17	1.8	8.30E-11	3.95E-12	1.13	5.97 ± 0.31	6.75 ± 0.28	C_6_H_12_O_7_	0.49%
Glucuronic acid	11.08	333.17	1.77	2.88E-10	1.57E-11	1.18	6.08 ± 0.46	7.18 ± 0.39	C_6_H_10_O_7_	0.52%
4-Hydroxybenzoic acid	9.27	223.12	1.74	3.76E-10	2.34E-11	1.20	4.66 ± 0.25	5.57 ± 0.51	C_7_H_6_O_3_	8.11%
Vanillylmandelic acid	10.71	297.16	1.7	3.76E-10	2.55E-11	1.16	4.72 ± 0.26	5.49 ± 0.38	C_9_H_10_O_5_	2.65%
Sucrose	14.41	361.19	1.72	5.08E-10	3.80E-11	1.16	5.81 ± 0.37	6.74 ± 0.40	C_12_H_22_O_11_	0.48%
2-Deoxytetronic acid	7.83	233.14	1.75	5.37E-10	4.38E-11	1.08	5.22 ± 0.20	5.65 ± 0.13	C_4_H_8_O_4_	0.43%
Arabitol	9.77	217.12	1.73	6.45E-09	5.70E-10	1.13	6.28 ± 0.41	7.07 ± 0.23	C_5_H_12_O_5_	1.26%
Indole-3-acetic acid	11.33	202.16	1.63	6.67E-09	6.36E-10	1.11	5.07 ± 0.26	5.61 ± 0.22	C_10_H_9_NO_2_	1.87%
Melezitose	15.55	361.21	1.58	1.84E-08	1.88E-09	1.23	4.54 ± 0.36	5.58 ± 0.66	C_18_H_32_O_16_	1.09%
Ribose	9.68	307.2	1.65	2.29E-08	2.49E-09	1.11	4.74 ± 0.26	5.24 ± 0.18	C_5_H_10_O_5_	0.80%
3-Aminopropionitrile	7.72	239.13	1.62	2.84E-08	3.28E-09	1.07	4.63 ± 0.12	4.97 ± 0.22	C_3_H_6_N_2_	1.81%
Guanidinosuccinate	11.46	444.19	1.59	1.62E-07	1.98E-08	1.29	3.96 ± 0.67	5.11 ± 0.44	C_5_H_9_N_3_O_4_	4.78%
Quinic acid	10.61	345.21	1.56	8.45E-07	1.09E-07	1.21	4.80 ± 0.53	5.81 ± 0.61	C_7_H_12_O_6_	0.49%
Mucic acid	11.18	318.18	1.47	9.39E-07	1.28E-07	1.19	5.20 ± 0.49	6.21 ± 0.67	C_6_H_10_O_8_	0.36%
Alloinositol	11.56	318.2	1.52	1.87E-06	2.68E-07	1.12	5.46 ± 0.38	6.10 ± 0.35	C_6_H_12_O_6_	0.15%
Lactose	14.7	204.11	1.45	1.97E-06	2.95E-07	1.13	5.12 ± 0.30	5.77 ± 0.49	C_12_H_22_O_11_	2.75%
Glucoheptonic acid	10.11	217.14	1.48	2.73E-06	4.27E-07	1.10	5.44 ± 0.35	5.98 ± 0.26	C_7_H_14_O_8_	1.32%
3-Phenyllactic acid	8.96	193.12	1.39	6.36E-06	1.04E-06	1.13	4.82 ± 0.37	5.45 ± 0.43	C_9_H_10_O_3_	2.30%
Glucose-1-phosphate	13.03	217.1	1.42	1.05E-05	1.78E-06	1.14	5.82 ± 0.43	6.66 ± 0.67	C_6_H_13_O_9_P	1.86%
Digalacturonic acid	13.7	217.11	1.22	1.24E-04	2.20E-05	1.14	4.59 ± 0.31	5.23 ± 0.68	C_12_H_18_O_13_	1.80%
5-6-Dihydrouracil	3.96	171.04	1.33	2.30E-04	4.22E-05	1.06	8.17 ± 0.49	8.70 ± 0.21	C_4_H_6_N_2_O_2_	9.09%
β-Alanine	6.46	146.05	1.23	2.64E-04	5.03E-05	1.05	5.73 ± 0.24	6.02 ± 0.32	C_3_H_7_NO_2_	5.00%
Pyrophosphate	9.44	451.13	1.26	2.93E-04	5.79E-05	1.07	6.26 ± 0.32	6.67 ± 0.32	C_8_H_20_O_7_P_2_	5.71%
Pipecolinic acid	7.11	156.14	1.12	4.60E-04	9.39E-05	−1.14	4.66 ± 0.52	4.10 ± 0.33	C_6_H_11_NO_2_	7.64%
Maltotriitol	15.05	361.13	1.13	1.49E-03	3.75E-04	1.07	5.62 ± 0.38	6.01 ± 0.30	C_18_H_34_O_16_	1.89%

Fold change: LDG/EDG when LDG > EDG; -EDG/LDG when EDG > LDG.

### 3.4 Diagnostic performances of the metabolites

**FIGURE 6:**
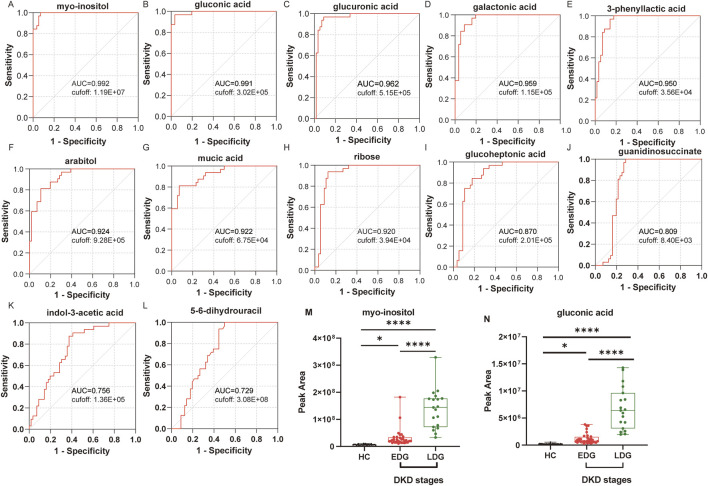
Receiver operating characteristics (ROC) curves of the diagnostic metabolites: **(A)** myoinositol, **(B)** gluconic acid, **(C)** glucuronic acid, **(D)** galactonic acid, **(E)** 3-phenyllactic acid, **(F)** arabitol, **(G)** mucic acid, **(H)** ribose, **(I)** glucoheptonic acid, **(J)** guanidinosuccinate, **(K)** indole-3-acetic acid, and **(L)** 5-6-dihydrouracil. **(M)** Peak area under the ROC curve (AUROC) values for myoinositol between the HC, EDG, and LDG groups. **(N)** Peak AUROC values for gluconic acid between the HC, EDG, and LDG groups (*p < 0.05; ****p < 0.0001).

To further assess the potential predictive powers of the identified biomarkers for DKD, an ROC analysis was conducted on the differential metabolites. Based on the results of the correlation analysis, 13 differential metabolites were selected as having correlations with all four clinical indicators. The ROC results showed that the area under the ROC curve (AUROC) values of these 13 metabolites were almost always high (above 0.85), indicating a high level of predictive ability. The ROC curves based on the OPLS-DA and Pearson correlation analysis were used to search for potential biomarkers for the diagnosis of DKD. Among the metabolites, myoinositol and gluconic acid had high sensitivity (>90%), specificity (>90%), and AUROC (>0.990) values, indicating that they exhibited stronger predictive abilities than the others; further, these predictions were accurate and sensitive, making them potential biomarkers for the diagnosis of DKD (Figures 6A–L).

Based on the heatmap results, both potential biomarkers were upregulated in the patients’ bodies. Further analysis was then conducted to examine the trends in the levels of these biomarkers in the EDG and LDG patients relative to the HCs. Compared to the HCs, the serum myoinositol levels were significantly elevated in the EDG patients (*p* < 0.05), while both myoinositol and gluconic acid levels were significantly higher in the LDG patients (*p* < 0.001) (Figures 6M, N). These findings suggest that myoinositol and gluconic acid levels increase and accumulate progressively as DKD advances.

## 4 Discussion

Early detection, diagnosis, and treatment of DKD, which is the most common microvascular complication of diabetes, remains a challenge to clinicians. It is well known that multisystem metabolism is altered in DKD (Pereira, et al., 2022). We reveal the serum metabolic profiles of DKD patients based on untargeted metabolomics and are committed to finding a suitable diagnostic method. Additionally, we identified two serum metabolites, namely, myoinositol and gluconic acid, which could serve as biomarkers of DKD. Previous studies have demonstrated that urinary inositol levels are associated with the eGFR and urine protein-to-creatinine ratio (UPCR), both of which are predictive of end-stage renal disease (ESRD) (Kwon et al., 2023). These findings align with our observations from the serum samples. Additionally, gluconic acid has been linked to the progression of CKD, further supporting the results of our study (Hong et al., 2024).

Myoinositol is a racemic compound of inositol and is considered to be a part of the vitamin B group (Croze and Soulage, 2013). It is involved in the serum metabolic pathways of ascorbate and aldarate metabolism as well as galactose metabolism. Ascorbate and aldarate metabolism is known to protect cells from oxidative damage; it also has a close relationship with the glucuronate pathway (Liang and Song, 2024; Peng et al., 2023). Galactose metabolism is an important carbohydrate metabolic pathway that is also known to be associated with cellular oxidative stress (Conte et al., 2021). Oxidative stress has been suggested as one of the possible pathogeneses of DKD, indicating that these two metabolic pathways may be related to the occurrence of DKD.

Most of the myoinositol in the body is synthesized in the kidneys, and the kidneys are the sole organs for myoinositol catabolism by myoinositol oxygenase; thus, the regulation of inositol cannot be separated from the kidneys (Kwon et al., 2023). The level of myoinositol in urine is considered to be closely related to insulin resistance. Moreover, depletion of myoinositol is believed to be related to the hemodynamic disorder of the kidneys in diabetes and even the direct cause of glomerulosclerosis and proteinuria (Bizzarri et al., 2017; Glass and Olefsky, 2012). Based on our findings, the serum myoinositol levels in DKD patients are higher than those in the HCs, and the AUROC of 0.992 reflects the accuracy of serum myoinositol level as a critical factor in the diagnosis of DKD. Based on the importance of the kidneys in the regulation of myoinositol as well as the results of the serum myoinositol levels observed across studies, myoinositol can be positively considered as a diagnostic biomarker of DKD.

Gluconic acid, also known as dextrogluconic acid, is an intermediate in the oxidative degradation of glucose in the body through the pentose phosphate pathway and is one of the oxidative stress markers. Gluconic acid has been associated with hyperglycemia and cytotoxic brain injury linked to oxidative stress (Ament et al., 2021; Fu et al., 2019). Gluconic acid is also associated with deterioration of renal function in patients with end-stage CKD, where it may be linked with the extent of kidney impairment, and oxidative stress leading to elevated plasma levels in patients with acute kidney injury (Cui et al., 2021). The significantly elevated gluconic acid levels observed in DKD patients in this study indicate obvious oxidative damage.

Glucuronic acid metabolism plays a critical role in cellular detoxification, extracellular matrix remodeling, and cell adhesion and migration. UDP-glucose 6-dehydrogenase (UGDH)-mediated production of UDP-glucuronic acid stabilizes TGFβR1 mRNA by promoting its binding to polypyrimidine tract-binding protein 3, thereby contributing to the activation of the TGFβ/*S*MAD signaling pathway. Inhibition of either UGDH or TGFβR1 is known to impair the metastasis of hepatocellular carcinoma metastasis (Gao et al., 2022). Both mucic acid and galactonic acid are involved in galactose metabolism, which has been strongly associated with diabetic nephropathy, consistent with our findings (Han et al., 2023). Additionally, increased urinary excretion of arabitol has been observed in children with CKD, which is also aligned with our observations with blood (Vanlede et al., 2015). Ribose reacts with hemoglobin to form glycosylated hemoglobin and enhance the glycosylation of serum protein, leading to the formation of advanced glycation end products (AGEs) that contribute to chronic diabetes complications, such as DKD (Tai et al., 2024). Guanidinosuccinate is a uremic toxin that inhibits renal platelets to disrupt hemostasis and promote inflammatory responses that are linked to microinflammation and activation of white blood cells. These differential metabolites are implicated in kidney diseases and diabetes-related nephropathy, and we hypothesize that they may also play similar roles in the pathogenesis of diabetic nephropathy (Cohen, 2007; Jubelirer, 1985).

Thus, using serum untargeted metabolomics approaches, we found that myoinositol and gluconic acid may provide precise measures for the diagnosis of DKD. Furthermore, elevated levels of these two metabolites may cause oxidative damage and lead to the occurrence of DKD. However, given that the levels of myoinositol and gluconic acid in the human body are often influenced by external factors, such as diet, these potential biomarkers may need to be measured during fasting in future studies to minimize such interference (Kijpaisalratana et al., 2023; Caputo et al., 2020).

## 5 Limitations

When interpreting the results of this study, one important limitation should be considered: the limited sample size in this study may impact the results to some extent. This study primarily focuses on differences in the metabolic profiles and development of diagnostic models; hence, it does not include validation of the biomarkers or further experimental investigations. To address these limitations, we are currently expanding the sample collection process with the goal of validating our findings. Our future research efforts will include proteomics, transcriptomics, and other approaches to enhance the accuracy and comprehensiveness of the findings.

## 6 Conclusion

An untargeted metabolomics approach based on GC-MS was successfully used to distinguish DKD patients from HCs in this study. Accordingly, a total of 68 distinguishable metabolites were identified, which are involved in arginine biosynthesis, ascorbate and aldarate metabolism, and galactose metabolism, among others. Metabolomics was also used to successfully distinguish between EDG and LDG patients as well as identify 31 distinguishable metabolites. We found a total of 30 differential metabolites involving the HC, EDG, and LDG groups simultaneously. To select biomarkers indicative of DKD, we used correlation analysis on four metrics (eGFR, BUN, SCr, and UACR) to identify 13 highly discriminative metabolites from among the 30 metabolites. ROC analysis was then used to demonstrate that the diagnostic performances of myoinositol and gluconic acid were greater than those of the other metabolites, with AUROC values of 0.992 and 0.991, respectively. Furthermore, these two metabolites are known to participate in the metabolic processes of DKD. Our work thus indicates a feasible diagnostic approach for detecting critical metabolites involved in DKD.

## Data Availability

The raw data supporting the conclusions of this article will be made available by the authors without undue reservation.
