# Supplementary material for: Serum metabolic profiling of patients with diabetic kidney disease based on gas chromatography-mass spectrometry
Source: Front Mol Biosci. 2025 Mar 17;12:1541440. doi: 10.3389/fmolb.2025.1541440 (PMC11955480; doi:10.3389/fmolb.2025.1541440)
Supplement: Supplementary file 1 [file Image1.pdf]

Serum Metabolic Profiling of Patients with Diabetic Kidney Disease Based on Gas Chromatography-mass Spectrometry

Xueyan Bian[1, †,*], Chenwen Wang[2,†], Majie Wang[3, 4,†], Ailing Yin[5], Jiayan Xu[1], Mijia Liu[1], Hui Wang[2], Yating Cao[5], Xin Huang[5], Chenxue Qin[1*], Ye Zhang[2*], Heming Yu[5*]

[1]Department of Nephrology, The First Affiliated Hospital of Ningbo University, Ningbo 315010, Zhejiang, China

[2]State Key Laboratory of Natural Medicines, School of Traditional Chinese Pharmacy, China Pharmaceutical University, Nanjing 211198, China

[3]Department of psychiatry, Affiliated Kangning Hospital of Ningbo University，Ningbo 315201，Zhejiang, China

[4]Department of psychiatry, Ningbo Kangning Hospital, Ningbo 315201，Zhejiang, China

[5]Nanjing Hospital of Chinese Medicine Affiliated to Nanjing University of Chinese Medicine, Nanjing 210023, China

*** Correspondence:**
njyhm6502@163.com (H.Y.); zhangye_cpu@cpu.edu.cn (Y.Z.); cxq2316@163.com (C.Q.); fyybianxueyan@nbu.edu.cn (X.B.)

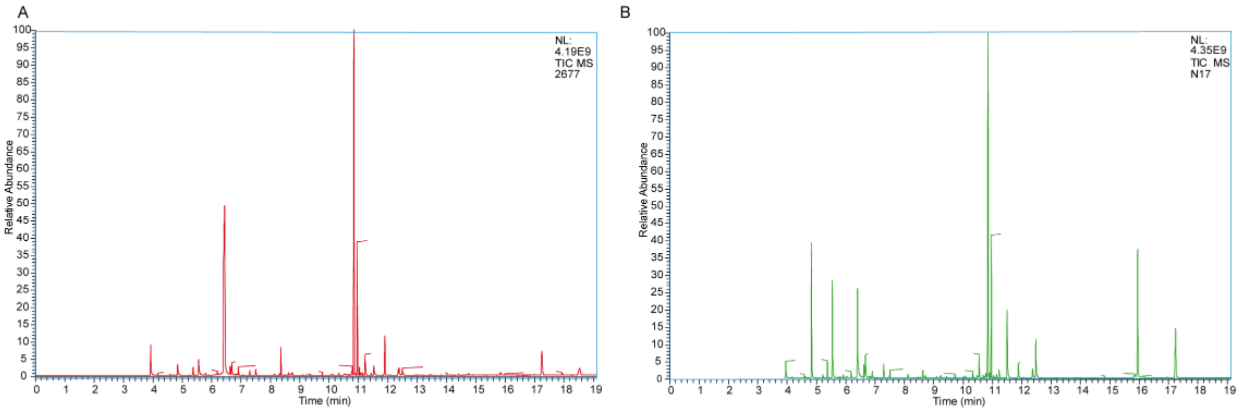

Figure S1. GC-MS total ion chromatograms of the DKD (A) and HC (B) groups.
